# Fluoride-Ion Batteries: On the Electrochemical Stability of Nanocrystalline La_0.9_Ba_0.1_F_2.9_ against Metal Electrodes

**DOI:** 10.3390/nano9111517

**Published:** 2019-10-25

**Authors:** Maria Gombotz, Veronika Pregartner, Ilie Hanzu, H. Martin R. Wilkening

**Affiliations:** 1 Institute for Chemistry and Technology of Materials, Technical Universtiy of Graz, 8010 Graz, Austria; 2 ALISTORE—European Research Institute, CNRS FR3104, Hub de l’Energie, Rue Baudelocque, 80039 Amiens, France

**Keywords:** solid fluoride electrolytes, ceramics, LaF3, fluorine-ion batteries, metal current collectors, electrochemical stability, cyclic voltammetry

## Abstract

Over the past years, ceramic fluorine ion conductors with high ionic conductivity have stepped into the limelight of materials research, as they may act as solid-state electrolytes in fluorine-ion batteries (FIBs). A factor of utmost importance, which has been left aside so far, is the electrochemical stability of these conductors with respect to both the voltage window and the active materials used. The compatibility with different current collector materials is important as well. In the course of this study, tysonite-type La${}_{0.9}$Ba${}_{0.1}$F${}_{2.9}$, which is one of the most important electrolyte in first-generation FIBs, was chosen as model substance to study its electrochemical stability against a series of metal electrodes viz. Pt, Au, Ni, Cu and Ag. To test anodic or cathodic degradation processes we carried out cyclic voltammetry (CV) measurements using a two-electrode set-up. We covered a voltage window ranging from −1 to 4 V, which is typical for FIBs, and investigated the change of the response of the CVs as a function of scan rate (2 mV/s to 0.1 V/s). It turned out that Cu is unstable in combination with La${}_{0.9}$Ba${}_{0.1}$F${}_{2.9}$, even before voltage was applied. The cells with Au and Pt electrodes show reactions during the CV scans; in the case of Au the irreversible changes seen in CV are accompanied by a change in color of the electrode as investigated by light microscopy. Ag and Ni electrodes seem to suffer from contact issues which, most likely, also originate from side reactions with the electrode material. The experiments show that the choice of current collectors in future FIBs will become an important topic if we are to develop long-lasting FIBs. Most likely, protecting layers between the composite electrode material and the metal current collector have to be developed to prevent any interdiffusion or electrochemical degradation processes.

## 1. Introduction

Lithium-ion batteries are by far the most widely used electrochemical energy storage systems. Since this battery technology starts to suffer from several issues, such as the exploitation of cobalt or nickel mines [1] in geopolitical disputed regions, alternative systems gain more and more importance. One of these alternate post-lithium systems, besides the development of sodium-ion batteries [2], relies on fluorine anions as the electrochemically active species.

The first explicit reference of a highly conducting F-bearing solid-state electrolyte, namely PbF${}_{2}$, was already in 1834 by Michael Faraday [3,4], who discovered ionic conduction in AgI and PbF${}_{2}$, see refs. [5,6] for an overview. In 1921, Carl Tubandt measured transference numbers of a series of solid electrolytes including PbF${}_{2}$ [7]. It then took several decades until 1971 when Baukal [8] and 1976 when Kennedy et al. [9] and Schoonman [10] constructed the first fluoride thin-film galvanic cells, see also refs. [11,12]. In 2011 Reddy et al. [13] revived the topic with a closer investigation of possible cathode materials [14,15,16], whereby La${}_{0.9}$Ba${}_{0.1}$F${}_{2.9}$, see also ref. [17], was chosen as solid state electrolyte due to its high conductivity of 2.8 × 10${}^{-4}$ S cm${}^{-1}$ at 160 ${}^{\circ }$C. Also polymer matrix [18] and liquid electrolytes [19] were investigated. Recently, several studies concerning various anode [20,21] and cathode materials [22] appeared; their suitability and performance have always been investigated in combination with La${}_{0.9}$Ba${}_{0.1}$F${}_{2.9}$ as the ceramic electrolyte of choice. To the best of our knowledge, no detailed studies concerning the electrochemical stability of solid-state fluoride electrolytes against metallic current collectors have been reported yet, neither of La${}_{0.9}$Ba${}_{0.1}$F${}_{2.9}$ nor of other F${}^{-}$ conductors such as Ba${}_{0.7}$Sb${}_{0.3}$F${}_{2.3}$, which is not stable against Ce as anode material [23], Ce${}_{1-x}$Sr${}_{x}$F${}_{3-x}$ (0 ≤*x* < 0.15) [24] or Sm${}_{1-x}$Ca${}_{x}$F${}_{3-x}$ (0.05 ≤ *x* < 0.15) [25]. Since a solid-state electrode must also contain a certain amount of (solid) electrolyte, it is important and meaningful to evaluate the stability of the electrolyte with respect to the current collector material.

**Figure 1:**
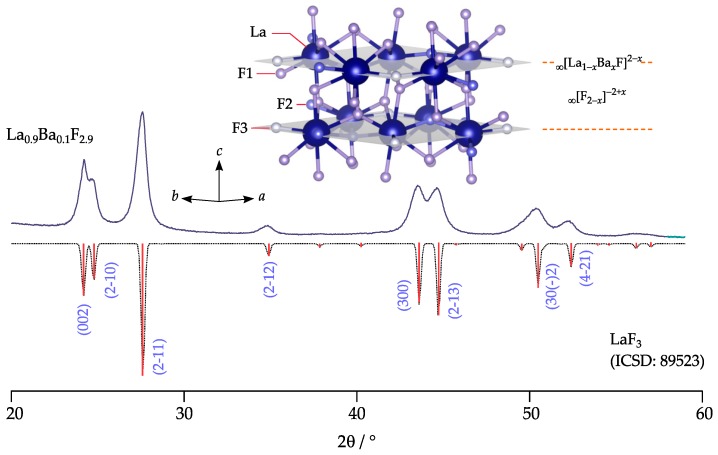
X-ray powder diffractogram of mechanosynthesized, nanocrystalline La${}_{0.9}$Ba${}_{0.1}$F${}_{2.9}$; the pattern was recorded at room temperature and ambient atmosphere. It is compared to that of LaF${}_{3}$, which serves as a reference (ICSD: 89523, $P\bar{3}c1$); LaF${}_{3}$ crystallizes with the well-known tysonite structure providing three different F sites labeled F1 (12g), F2 (4d) and F3 (2a). Each site is fully occupied by fluorine anions. F1 anions reside in the ${}_{\infty }{\left[F_{2-x}\right]}^{-2+x}$ interslabs and are located in distorted La${}_{4}$ tetrahedra.

In the course of this study, the electrochemical stability of nanocrystalline La${}_{0.9}$Ba${}_{0.1}$F${}_{2.9}$, synthesized following a mechanochemical route, was examined by cyclic voltammetry (CV). Prior to our stability tests we checked the purity of the tysonite phase via X-ray powder diffraction and carried out electrochemical impedance spectroscopy (EIS) to determine its F ionic conductivity. The crystal structure of La${}_{0.9}$Ba${}_{0.1}$F${}_{2.9}$ is illustrated in Figure 1. Here, we investigated the electrochemical stability of La${}_{0.9}$Ba${}_{0.1}$F${}_{2.9}$ in combination with various metal electrodes namely Cu, Ag, Au, Pt and Ni, which were applied to the compacted electrolyte pellets by direct current sputtering.

Usually, electrochemical stability of electrolytes is tested via CV in a three electrode set-up. As fluorine is not solid at ambient conditions, it cannot easily serve as a reference electrode, which makes the interpretation of the CV results obtained from a two electrode set-up, as applied here, rather difficult [26]. We also varied the size of the working electrode (WE); diameters were varied from 2.5 to 8 mm, to find out whether it has any influence on the results. Our CV measurements cover a voltage window from −1 to 4 V. This voltage window is similar to that used in earlier reports (1 to 3.5 V [13] and 0 to 3.5 V [22]). Compared to a recently published study [27] on La${}_{0.9}$Ba${}_{0.1}$F${}_{2.9}$, where we carried out CV at a rate of 100 mV/S, here we reduced the scan rate to 2 mV/s. Screening of five different metal electrode materials offers a quite diverse picture and highlights the reactivity of fluorine-containing electrolytes, as in none of the electrolyte/electrode configurations a stable electrochemical state could be achieved under the conditions mentioned above.

## 2. Materials and Methods

### 2.1. Mechanochemical Synthesis

Nanostructured La${}_{0.9}$Ba${}_{0.1}$F${}_{2.9}$ was mechanosynthesized with the help of a high-energy planetary mill (Fritsch Pulverisette 7 Premium line, Fritsch GmbH, Idar-Oberstein, DE). For this purpose, stoichiometric amounts of the educts viz. LaF${}_{3}$ (99.99%, Alfa Aesar, Kandel, DE) and BaF${}_{2}$ (99.99%, Sigma Aldrich, Darmstadt, DE) were loaded into a ZrO${}_{2}$ milling beaker with a volume of 45 mL. We added 180 balls made of the same material; the diameter of each milling ball was 5 mm. The milling procedure was carried out at a rotation speed of 600 rpm; the mixture was milled for 10 h whereby 15 min milling was followed by a break of 15 min to allow cooling of the mixture and the beaker. Loading as well as unloading of the beakers was strictly carried out under inert atmosphere; we used an Ar-filled glovebox (O${}_{2}$, H${}_{2}$O < 0.5 ppm) to avoid any contamination by water vapor or moisture.

### 2.2. X-Ray Powder Diffraction

The powdered sample was analyzed by X-ray powder diffraction (XRPD) to ensure that the educts have fully been transformed to La${}_{0.9}$Ba${}_{0.1}$F${}_{2.9}$ crystallizing, as LaF${}_{3}$ does, in the tysonite phase. Diffractogramms were recorded with a Bruker D8 Advance diffractometer operating with Bragg Brentano geometry and CuKα radiation (1.5406 Å). XRPD reflections were recorded over a 2θ range of 10 to 100${}^{\circ }$2θ (stepsize 0.02${}^{\circ }$2θ, 1 s measuring time per step). With the aid of the program X-PertHighScorePlus (PANalytical, Malvern, UK) we analyzed the diffractogramms according to the refinement procedure introduced by Rietveld.

### 2.3. Broadband Impedance Spectroscopy

The ionic conductivity of the as-prepared La${}_{0.9}$Ba${}_{0.1}$F${}_{2.9}$ was checked by solid-state impedance spectroscopy. Hence, the powder samples were pressed to cylindrical pellets with a diameter of 5 mm and a thickness of ca. 1 mm; we used a press of P. O. Weber and applied a uniaxial force of 0.5 t. Ion blocking Pt electrodes with a thickness of 100 nm were applied on both sides of the pellet by sputtering (Leica sputter device (EM SCD050, Leica Microsystems, Wetzlar, DE)). Impedance data were recorded with a Novocontrol Concept 80 broadband spectrometer in combination with an active BDS 1200 cell [28] and a ZGS interface (Novocontrol). Impedance data were recorded over a frequency range from 10${}^{-2}$ Hz to 10${}^{7}$ Hz at temperatures ranging from 20 to 200 ${}^{\circ }$C. A QUATRO cryo-system (Novocontrol, Montabaur, DE) controlled the temperature of all measurements, which were carried out under a constant flow of dry, freshly evaporated N${}_{2}$ gas.

### 2.4. Cyclic Voltammetry

**Figure A1:**
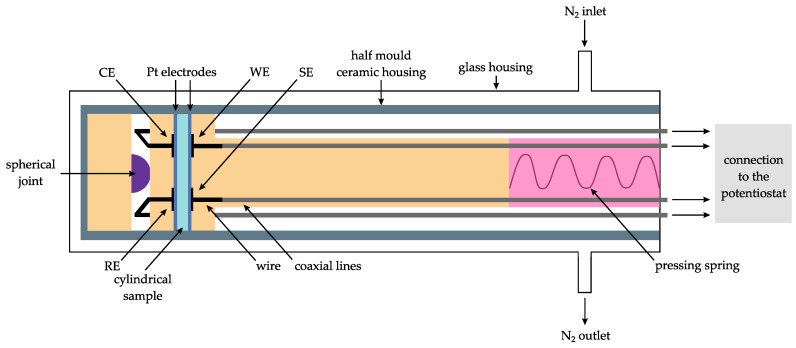
Scematic drawing of the cell set-up for conducting solid state Cyclic Voltammetry in a two-electrode set-up.

For all-solid-state cyclic voltammetry measurements approximately 120 mg of the sample powder were pressed into cylindrical pellets with a diameter of 8 mm. After uni-axial pressing (0.4 t) the thickness of the pellets ranged from 0.9 to 1 mm. As reference and counter electrode (CE) different materials of high purity (Au, Ag, Cu, Pt, Ni) were sputtered on one complete side of the pellets. We studied pellets equipped with sputtered working electrodes (Leica, EM SCD050) of the following diameters *d*: 2.5, 4, 6 and 8 mm. Again, all preparation steps were carried out in a glovebox filled with Ar (H${}_{2}$O, O${}_{2}$ < 0.5 ppm). The sample chamber of the sputter device was purged with Ar 5.0 for five times before the sputter process was started to avoid any contamination with moisture or O${}_{2}$. Cyclic voltammetry was performed with a Parstat MC potentiostat (Princeton Applied Research) equipped with a low-current option. We carried out all measurements at a temperature of 200 ${}^{\circ }$C in a cell, which was continuously purged with N${}_{2}$. Scanning rates ranged from 0.002 to 0.1 V/s whereby the voltage was varied from −1 V to 4 V. A schematic drawing of the cell set-up is shown in Figure A1.

### 2.5. Scanning Electron Microscopy

The surface of the pellets (Ag and Au as electrode, working electrode d=2.5mm) before and after CV were investigated by digital light microscopy (Di-Li Digital-Mikroskop, TCapture) and by scanning electron microscopy (SEM, VEGA3 TESCAN), in both the secondary electron (SE) and backscattered electron (BSE) mode. SEM was combined with energy dispersive X-ray spectroscopy (EDX) to analyze the elemental distribution at the electrode/electrolyte interface. IGOR Pro (Wavemetrics) software was used for data analysis.

## 3. Results and Discussion

**Figure 2:**
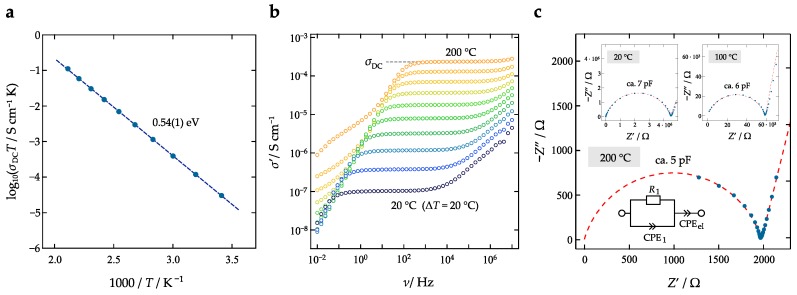
(**a**) Arrhenius Plot of the ionic conductivity $\sigma_{\mathrm{DC}}$ multiplied with absolute temperature *T* vs. 1000/T of nanocrystalline La${}_{0.9}$Ba${}_{0.1}$F${}_{2.9}$ that was prepared by a mechanochemical approach. (**b**) Conductivity isotherms of La${}_{0.9}$Ba${}_{0.1}$F${}_{2.9}$ starting from 20 ${}^{\circ }$C and ending at 200 ${}^{\circ }$C, which was the highest temperature where we measured conductivities. (**c**) Nyquist representation of the complex impedance data of La${}_{0.9}$Ba${}_{0.1}$F${}_{2.9}$; the curve refers to a temperature of 200 ${}^{\circ }$C and shows the imaginary part of the complex impedance $-Z^{\prime \prime }$ plotted vs. the real part $Z^{\prime }$. The inset shows additionally the Nyquist representation at temperatures of 20 ${}^{\circ }$C and 100 ${}^{\circ }$C. The spike at low frequencies represents electrode polarization effects originating from the ion-blocking electrodes used. The whole location curve was parameterized with the equivalent circuit shown in the inset. It consists of a resistor $R_{1}$ connected in parallel to a constant phase element (CPE); this $R_{1}$-CPE${}_{1}$ element represents the overall (bulk) semicircle and leads to an associated capacitance *C* [32,33] of ∼5 pF, which is a typical value for electrical relaxation taking place in the interior regions of the grains [29]. Another CPE element, connected in series, was used to approximate electrode polarization.

The phase purity of the mechanosynthesized La${}_{0.9}$Ba${}_{0.1}$F${}_{2.9}$ sample was verified by XRPD. The diffractogram, which is depicted in Figure 1, is composed of broad reflections due to the small grain size obtained after the milling process. According to Scherrer’s equation we estimate that the mean crystallite diameter is in the order of 10 to 20 nm; a similar result has been reported recently for the same material [27]. XRPD reveals that the solid solution crystallizes with the tysonite structure of LaF${}_{3}$ (ICSD: 89523). Lattice constants, slightly deviating from that of Ba-free LaF${}_{3}$, point to successful incorporation of the Ba${}^{2+}$ ions. Conductivity spectroscopy revealed that, as expected for the electrolyte La${}_{0.9}$Ba${}_{0.1}$F${}_{2.9}$, the ionic conductivity at a temperature of 200 ${}^{\circ }$C was $\sigma^{\prime }\left(\nu \to 0\right)\equiv \sigma_{\mathrm{DC}}=2.36\times 10^{-4}$ S/cm. This value can be directly read off from the conductivity isotherms, which are shown in Figure 2 alongside with the Arrhenius plot of $\sigma_{\mathrm{DC}}T$ vs. 1000/T. For selected temperatures, viz. for 20, 100 and 200 ${}^{\circ }$C the data are also shown using the representation according to Nyquist [29]. The conductivity isotherms shown here perfectly agree with those published earlier by our group, see Breuer et al. [27]. For a general discussion and interpretation of conductivity data we refer to the literature [29,30,31].

To evaluate the compatibility of the electrolyte with a range of current collector materials, the various metals were applied on both sides of the cylindrical pellets by sputtering. As the reaction of interest is supposed to occur at the working electrode, we took into account that the current flow at the working electrode should, in principle, never be limited by the current flow passing through the reference/counter electrode. In classical liquid-based electrochemistry, this condition is achieved by using a counter electrode with a much larger surface than that of the working electrode. This condition is much more difficult to achieve in the solid state. However, here we investigated the influence of the size of the working electrode and varied it from 2.5, 4, 6 to 8 mm; the latter diameter corresponds to the full size of the surface of the pellet.

**Figure A2:**
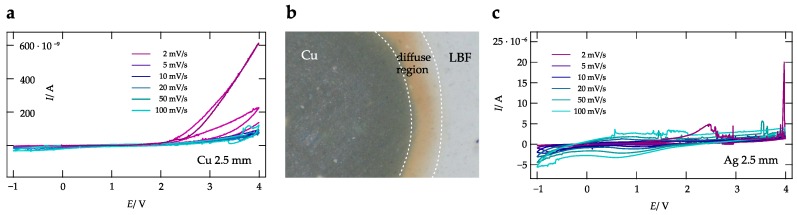
CV of a La${}_{0.9}$Ba${}_{0.1}$F${}_{2.9}$ cell with Cu (**a**) and Ag (**c**) as electrode material (d=2.5mm, 0.002 V/s to 0.1 V/s, 200 ${}^{\circ }$C). (**b**) Light microscopy image of a Cu cell (d=2.5mm) *after* CV. At the Cu|electrolyte interface a diffusive region with orange color appears.

Except for Cu, all electrode materials were sputtered with success and did not show any direct reaction with the electrolyte. However, in the case of the Cu electrode we noticed a silver colored surface directly after the sputtering process. Irrespective of this change in color, we took a pellet with a working electrode of 2.5 mm in diameter, which was subjected to CV. CV measurements resulted in a noisy signal due to contact problems, as it is shown in Figure A2a. At a potential *E* larger than 2 V we notice electrochemical reactions, at least at very low scan rates of 2 mV/s. The corresponding maximum current amplitude is relatively small and turned out to be in the order of 0.6μA at E=4V. Additionally, inspection by light microscopy revealed a diffuse region with coppery color between the actual electrode and pure La${}_{0.9}$Ba${}_{0.1}$F${}_{2.9}$, see Figure A2b.

**Figure 3:**
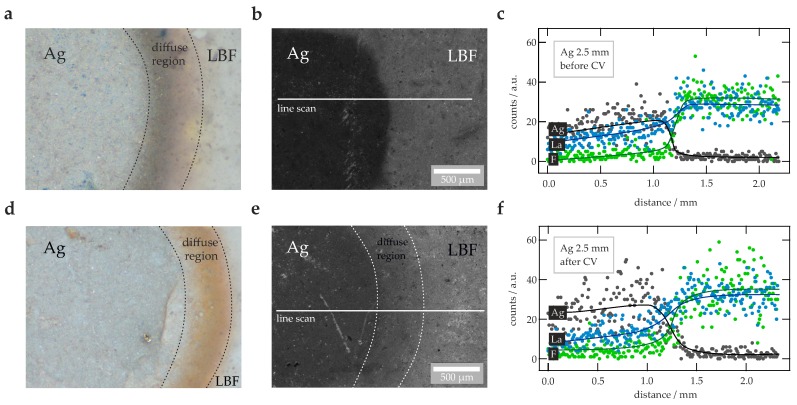
(**a**) Images acquired by digital light microscopy of a sample pellet with Ag as working electrode (d=2.5mm) *before* the cyclic voltammetry (CV) measurements have been carried out. (**b**) SEM image (secondary electron (SE) detector) of this pellet before CV. The horizontal line indicates the run of the energy dispersive X-ray spectroscopy (EDX) line scan. (**c**) EDX line scan across the surface of the pellet, as depicted in (**b**), for the elements F, Ag and La. The lines are only a guide for the eye. (**d**) Image obtained via digital light microscopy of a sample pellet with Ag working electrode *after* CV. (**e**) SEM image (SE detector) of the sample pellet with Ag as working electrode, the image was taken after the CV measurements. The horizontal line indicates the run of the (EDX) line scan. (**f**) EDX line scan across the surface of the pellet, as depicted in (b), for the elements F, Ag and La, respectively The lines are drawn to guide the eye.

Similar results were found for the Ag electrode. From visual inspection the electrode appeared to have not reacted with the electrolyte; CV, however, points to irreversible changes, as is documented in Figure A2c. During cycling the electrode also suffered from contact issues and changed color from silvery to orange. Below E=4V the current amplitude did not exceed values above 5μA; thus larger as in the case of the Cu electrode. Hence, we investigated the electrode and the surface of the electrolyte via light microscopy, SEM and EDX line scanning (see Figure 3). Changes in color at the Ag | La${}_{0.9}$Ba${}_{0.1}$F${}_{2.9}$ interface are clearly visible in Figure 3, which shows SEM images of the surface before (Figure 3a) and after cycling (Figure 3d). SEM images taken in SE mode, see (Figure 3b) and (Figure 3e), indicate that the original clear border between the electrode and the ternary fluoride La${}_{0.9}$Ba${}_{0.1}$F${}_{2.9}$ became more diffuse. Presumably, migration of Ag is responsible for this observation. To prove this assumption EDX linescans across the interface (as indicated by the horizontal bars in (Figure 3b) and (Figure 3e)) were performed. The results from before (Figure 3c) and after CV (Figure 3f) indeed reveal a trend in Ag distribution. While the concentration of Ag on the reference surface falls away sharply at the Ag | La${}_{0.9}$Ba${}_{0.1}$F${}_{2.9}$ interface, after the CV measurements it does not. Therefore, we conclude that Ag migrates in the material, visibly over the surface during cycling. Ag migration leads to irreversible changes at this interface.

**Figure 4:**
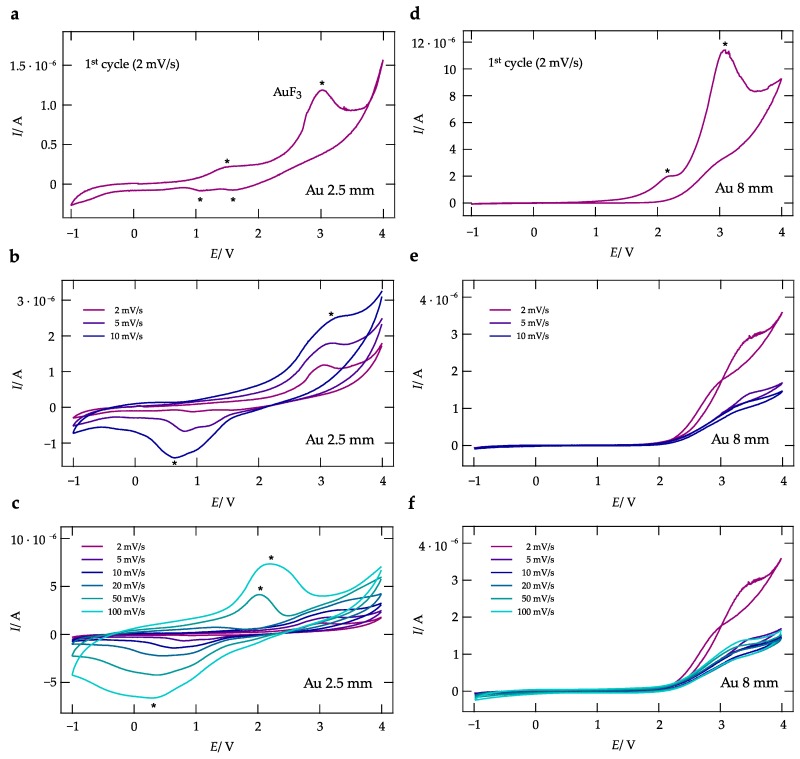
Cyclic voltamogram of cells with La${}_{0.9}$Ba${}_{0.1}$F${}_{2.9}$ as solid electrolyte and Au working as electrode metal. The CVs were recorded at a temperature of 200 ${}^{\circ }$C, with scan rates ranging from 2 mV/s up to 0.1 V/s; working electrode diameters are 2.5 mm (**a**–**c**) and 8 mm (**d**–**f**). The current response of the first cycles are shown in (**a**,**d**).

**Figure 5:**
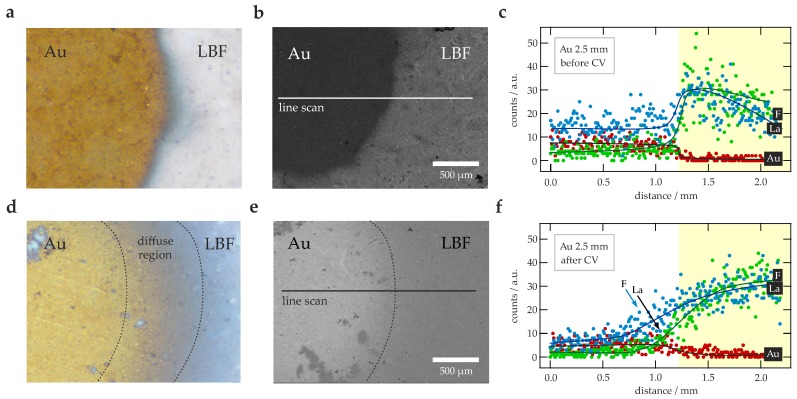
(**a**) Digital light microscopy image of a sample pellet with Au working electrode (d=2.5mm) before the CV measurements were carried out. (**b**) SEM image (SE detector) of the sample pellet with the Au working electrode *before* CV. The horizontal line indicates the run of the EDX line scan. (**c**) EDX line scan across the surface of the pellet, as depicted in (**b**), for the elements F, Au and La. The lines are only a guide to the eye. (**d**) Image taken by digital light microscopy of the cell *after* CV. (**e**) SEM image (backscattered electron (BSE) detector) of the cell with Au electrodes *after* the CV measurements. The horizontal line indicates the run of the EDX line scan. (**f**) EDX line scan across the surface of the pellet, as depicted in (e), for the elements F, Au and La. The lines serve as a guide to the eye.

In Figure 4a,b CVs of the symmetrical cell Au | La${}_{0.9}$Ba${}_{0.1}$F${}_{2.9}$ | Au, recorded in a voltage window of −1 to 4 V and scan rates ranging from 2 mV/s to 0.1 V/s, are shown. The corresponding images from light microscopy are depicted in Figure 5. In the cyclic voltamogram with the WE having a size of 2.5 mm, see (a), distinct anodic and cathodic peaks are visible at a scan rate of 2 mV/s. Two anodic peaks (positive direction of scanning) are clearly visible and also two less apparent cathodic peaks show up in the negative direction of scanning. The main anodic peak at E=3 V, whose counterpart is visible at a faster scan rate of 10 mV/s at ca. E=0.6 V. Most likely, these peaks reveal the formation (and decomposition) of AuF${}_{3}$ at the Au | La${}_{0.9}$Ba${}_{0.1}$F${}_{2.9}$ interfaces. With increasing cycling, i.e., with increasing Au interdiffusion (see below), the shape of the CVs changes. At scan rates of 50 and 100 mV/s anodic and cathodic peaks at lower voltages appear indicating the irreversibly change of the interface on its electrochemical behavior. The maximum current amplitude that is reached at a scan rate of 100 mV/s is approximately 8μA at ca. E=2.1 V.

**Figure A3:**
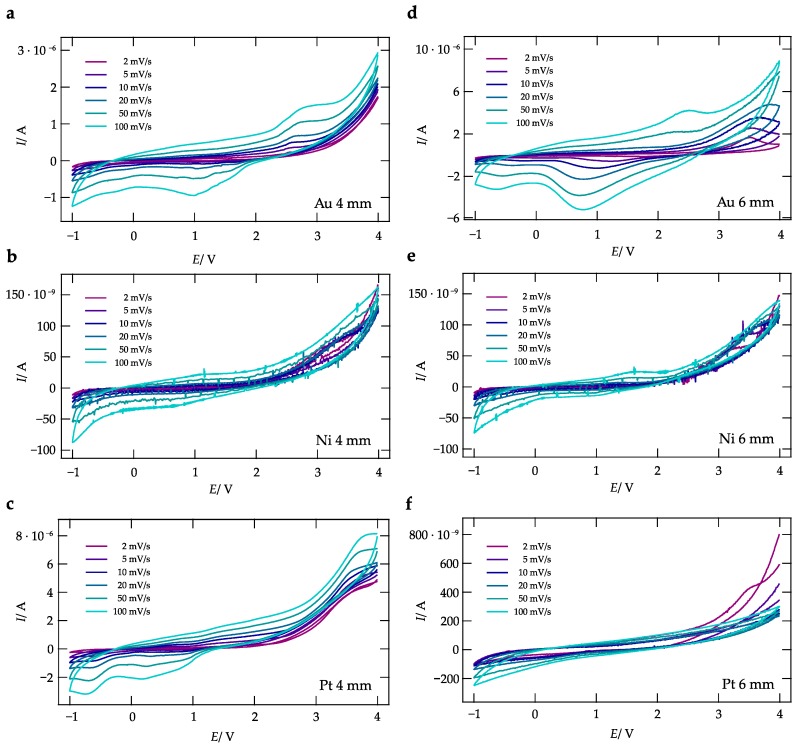
CVs for symmetric La${}_{0.9}$Ba${}_{0.1}$F${}_{2.9}$ cells (0.002 V/s to 0.1 V/s, 200 ${}^{\circ }$C) with Au electrodes (WE d=4mm and d=6mm see (**a**,**d**)), Ni electrodes (WE d=4mm and d=6mm see (**e**,**b**)), and Pt electrodes (WE d=4mm and d=6mm see (**c**,**f**)).

The first cycle of a pellet with a diameter of the working electrode of 8 mm is depicted in Figure 4f. When compared with the CV recorded using a disc with d=2.5mm, similarities can be seen. Again, two peaks in the positive direction occur. In contrast, no cathodic peaks are visible in this case. This trend continues when going to higher scan rates, see Figure 4e,f, as the curves do not reveal any peaks at *E* lower than 2 V but current responses as high as 3μA when *E* reaches 3.5 V. CV’s for WE diameters of 4 and 6 mm are shown for the sake of brevity in the Appendix A, see Figure A3a,d, as no major differences compared to a WE diameter of 2.5 mm are recognizable. If we take for example a look on Figure 4, where Au is used as electrode material, a small scan rate results in a small current for a WE diameter of 2.5 mm and the current is increasing with the scan rate. The opposite trend is, however, visible for a WE diameter of 8 mm; the corresponding current is decreasing with the scan rate. The difference in behavior between the 2.5 mm WE and the 8 mm WE can be traced back to the influence of the CE. As mentioned in the experimental section, the CE electrode is covering one entire side of the pellet (8 mm in diameter) and only the diameter of the WE is varied. For a 2.5 mm WE with an 8 mm CE the ratio between the surface areas is higher than 1:10. A large surface area of the CE with respect to the WE is necessary whenever a 2-electrode cell configurations is used to ensure that the current through the cell is limited by the WE, then the influence of the CE would be small. This is, however, not the case when 8 mm working electrodes are used, the WE:CE surface area ratio is 1:1. Thus, in this case, the CE would show a significant influence on the current response of the cell, as seen in Figure 4.

Similarly to our observations with Ag as electrode, investigations of the Au | La${}_{0.9}$Ba${}_{0.1}$F${}_{2.9}$ interface by light microscopy revealed a color change at the border of the two phases (see Figure 5a,d). The change is seen after the CV measurements have been carried out, hence during cycling Au interdiffuses into the electrolyte phase. This observation is independent of the diameter of the working electrode used. Au interdiffusion is also supported by SEM images, using either the SE or the BSE detector. The originally clear border between the materials (Figure 5b) has become blurred (Figure 5e). EDX line scans across the border carried before and after cycling (Figure 5c,f) underpin our conclusion of Au migration as the distribution function of the elements changes from a step-like shape to a sigmoid-like one. In particular, also the F concentration profile (and the La profile as well) smears out, which indicates the formation of binary Au-F compounds, possibly tysonite-type La${}_{1-x}$Au${}_{x}$F${}_{3}$.

**Figure 6:**
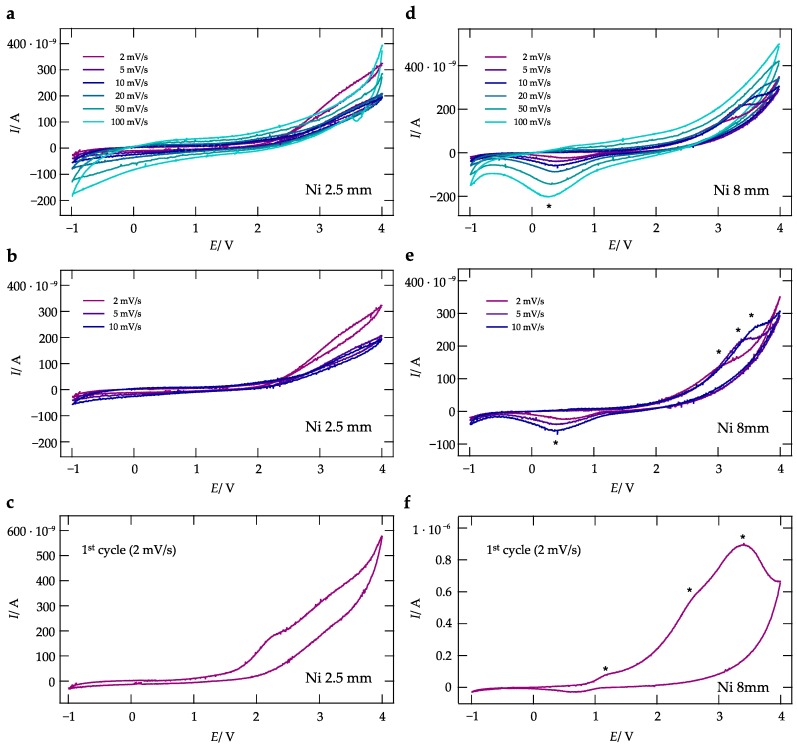
CVs of symmetrical cells with La${}_{0.9}$Ba${}_{0.1}$F${}_{2.9}$ as electrolyte and Ni as electrode material. CVs were recorded at a temperature of 200 ${}^{\circ }$C; scan rates ranged from 2 mV/s to to 0.1 V/s. The diameter of the working electrodes was either 2.5 mm (**a**–**c**) or 8 mm (**d**–**f**).

The corresponding CVs of the sandwich cells with Ni as electrode material are shown in Figure 6. As evidenced by the slightly noisy signal, the measurements suffer a bit from contact issues.

The CV measurements on a cell with a 2.5 mm thick WE do not reveal any distinct current peaks. As in the case of the cells with Au electrodes, the diameter of the WE, see the curves obtained with cells equipped with a WE having a diameter of 8 mm, yields somewhat different results at high scan rates; for instance, we see that at a scan rate of 0.1 V/s a prominent current peak appears at 0.2 V, which is, however, absent for WEs with a diameter of only 2.5 mm. This peak is also seen at lower scan rates. Correspondingly, peaks with positive current and low intensity show up at ca. 3.4 V, at least at lower scan rates. The latter peak dominates the current response also during the first cycle (see Figure 6d,f). It has shoulders at 2.5 V and 1.2 V, respectively. Note that the current amplitude turned out to be much larger for the very first cycle, indicating irreversible reduction/oxidation processes taking place at the Ni|La${}_{0.9}$Ba${}_{0.1}$F${}_{2.9}$ interface. The formation of NiF${}_{2}$ passive films on the surface of Ni is known and it seems that such a layer is also forming here. Cells with WE having diameters of 4 and 6 mm, respectively, result in basically identical CVs as seen for the cells with a 2 mm WE. The corresponding responses are shown in Figure A3b,e.

**Figure 7:**
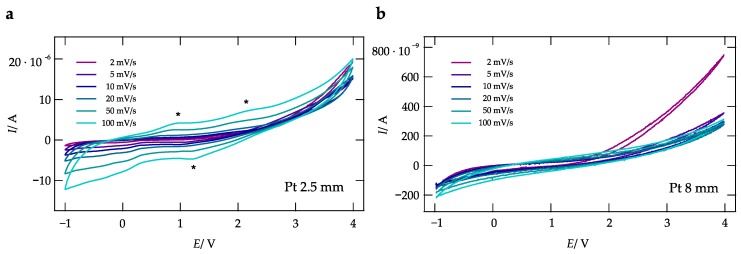
CVs of a La${}_{0.9}$Ba${}_{0.1}$F${}_{2.9}$ cell with two Pt electrodes. The diameter of the WE was either 2.5 mm (**a**) or (**b**) 8 mm. CVs were recorded at scan rates ranging from 0.002 V/s to 0.1 V/s at 200 ${}^{\circ }$C.

Finally, in Figure 7, CVs of Pt|La${}_{0.9}$Ba${}_{0.1}$F${}_{2.9}$|Pt cells with a WE having a diameter of 2.5 mm (Figure 7a) or 8 mm (Figure 7b) are shown.

Again, scan rates were varied to range from 2 mV/s to 0.1 V/s. For cells with a WE having a diameter of 8 mm no distinct oxidation/reduction peaks are visible. This observation is in contrast to the situation of the cell with a 2.5 mm WE. Two shallow peaks with positive currents and at least one in the direction of negative current are seen. For the sake of completeness, the results from CV on cells with WE having diameters of 4 mm and 6 mm are shown in Figure A3c,f.

## 4. Conclusions and Outlook

We tested the electrochemical stability of several metal electrodes that might work as current collector materials in the first generation of fluorine-ion batteries. We see that most metals tested are not in stable contact with the studied nanocrystalline ionic conductor La${}_{0.9}$Ba${}_{0.1}$F${}_{2.9}$. While Cu reacts on contact, also Ag, Au, Pt and Ni undergo complex electrochemical reactions showing instabilities at the interface between the metal current collector and the ceramic electrolyte. In addition, we also see a significant migration of the metals. This diffusion processes point toward the necessity of developing and using diffusion barrier layers in prospective fluoride-ion systems. While all the systems undergo degradation upon subsequent cycles in CV experiments, we also see that Ni is very likely the most efficient metal that is able to form a passivating layer at the metal|electrolyte interface.

## Abbreviations

The following abbreviations are used in this manuscript:

FIB	Fluorine Ion Battery
CV	Cyclic Voltammetry
EIS	Electrochemical Impedance Spectroscopy
SEM	Scanning Electron Microscopy
CE	Counter Electrode
WE	Working Electrode
EDX	Energy-Dispersive X-ray Spectroscopy
SE	Secondary Electrons
BSE	Backscattered Electrons
